# Origins of Common Neural Inputs to Different Compartments of the Extensor Digitorum Communis Muscle

**DOI:** 10.1038/s41598-017-14555-x

**Published:** 2017-10-24

**Authors:** Chenyun Dai, Henry Shin, Bradley Davis, Xiaogang Hu

**Affiliations:** Joint Department of Biomedical Engineering, University of North Carolina at Chapel Hill and North Carolina State University, NC, 27599 USA

## Abstract

The extensor digitorum communis (EDC) is a multi-compartment muscle that allows dexterous extension of the four digits. However, the level of common input shared across different compartments of this muscle is not well understood. We seek to systematically characterize the common and independent neural input, originated from different levels of the central nervous system, to the different compartments. A motor unit (MU) coherence analysis was used to capture the different sources of common and independent input, by quantifying the coherence of MU discharge between different compartments. The MU activities were obtained from decomposition of surface electromyogram recordings. Our results showed that the MU coherence across different muscle compartments accounted for only a small proportion (<20%) of the total input in the alpha (5–12 Hz) and beta (15–30 Hz) bands, but was a major driver (>60%) in the delta (1–4 Hz) band. Additionally, cross-compartment coherence between the middle and ring-little fingers tended to be higher as compared with other finger combinations. Overall, the common input shared across different fingers are found to be at low to moderate levels, in comparison with the total input, which allows dexterous control of individual digits with some degree of coordinated control of multiple digits.

## Introduction

The extensor digitorum communis (EDC) is a multi-compartment muscle controlling the extension of the four fingers (index, middle, ring and little). The individual compartment of this multi-tendinous muscle is considered to be controlled by subpopulations of a motoneuron pool, thereby realizing individualized digit control. Although a healthy individual has highly dexterous regulation of each individual finger, completely independent movement cannot be achieved^1–3^. These coupled finger movements are a result of both mechanical coupling and a shared common neural input^4^. Specifically, different alpha motor neurons innervating their conjoint muscle compartments of the EDC receive both unique and common synaptic input with spinal and supraspinal origins. Even though direct measurement of the neural input to the EDC of humans is not readily accessible, this common input can be estimated indirectly through a cross-correlation analysis between the firing times of different motor unit (MU) spike trains^5–7^.

Earlier studies have focused on the estimation of the time-domain common input within a muscle^8,9^. One previous study on the EDC muscle^10^ have used an approach combining cross-correlation histograms and an index of common input strength to evaluate discharge synchronization between MU pairs. The results show that the neighboring compartments share a moderate level of common input, which is lower than the common input within a compartment. However, we still have little knowledge regarding the origins of the common input, whether it arises from spindle activities, common modulation of the mean firing rate, or supraspinal common input. Additionally, the relative contribution between shared and separated common drive has not been systematically quantified during EDC muscle activation.

To address this issue, we adopted a MU discharge coherence analysis^11–14^ in the frequency-domain, in order to capture the different origins of common neural input both within and across compartments of the EDC muscle. The MU discharge coherence analysis allows us to quantify the degree of correlation at different frequency bands, which can reflect the different levels of physiological origins^5,13,15^. Specifically, previous studies have established that the coherence spectrum under 60 Hz contains most of the neural control information which can be divided into four frequency bands; namely the delta band (1–4 Hz), alpha band (5–12 Hz), beta band (15–30 Hz) and gamma band (30–60 Hz). Each bandwidth of synchronization represents a specific origin. The delta band is associated with the common modulation of mean firing rates^16^. The alpha band is believed to reflect some of the muscle spindle activity, resulting from the spinal reflex loop^17,18^. The beta band represents cortical and subcortical processes^19,20^. Lastly, the gamma band represents primarily cortical activities and also subcortical activities to some extent^19,21^, especially during dynamically changing muscle contractions^22^. Using a variation of this approach, termed partial coherence analysis^23,24^, a recent study^25^ has explored the neural input distribution of two synergistic muscles— vastus lateralis and vastus medialis muscles during isometric knee extension. In contrast to the earlier study on the EDC muscle^10^, their results reveal that the shared common neural input between muscles are the primary driver of these synergistic thigh muscles. The outcomes of our study could address these inconsistent findings, arising either from the different muscle groups (fine controlled finger muscle vs. leg muscles), or from the different analytical approaches that quantified the common input.

In our study, surface electromyogram (sEMG) signals of different compartmental regions were recorded during isometric voluntary contraction of the EDC muscle. A sEMG decomposition algorithm^26^ was used to decompose the EMG signals into constituent MU action potential trains, providing the discharge event times of individual MUs. The coherence of MU spike trains was evaluated both within a compartment and across different compartments. Our main findings reveal that the shared common input across different compartment pairs of the EDC only involve a small portion (<20%) of the corresponding total neural input in the alpha (5–12 Hz) and beta (15–30 Hz) bands. In contrast, the cross-compartment coherence in the delta (1–4 Hz) band had major contribution (>60%) to the total neural drive during common modulation of the firing rate. Lastly, we also found that the shared common input between the middle and ring-little fingers was higher than any other pairs, indicating less independent control of middle and ring-little fingers.

## Methods

### Experimental Apparatus and Data Collection

#### Subjects

Experimental data from eight healthy subjects (6 male, 2 female; between 19 to 35 years of age) was obtained. Each subject provided written informed consent. The protocols were approved by the Institutional Review Board at the University of North Carolina at Chapel Hill. All methods were performed in accordance with the relevant guidelines and regulations.

#### EMG Electrode Placement

**Figure 1:**
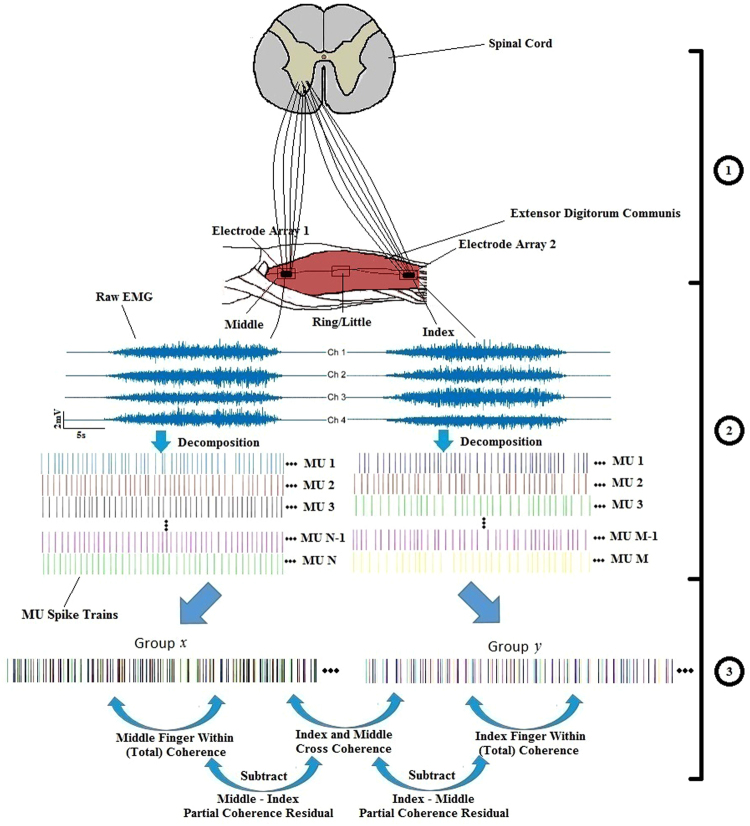
Block diagram of the data acquisition and analysis. Part 1 presents the high level neural control scheme. Part 2 presents data collection and EMG decomposition process. Part 3 presents the general coherence analysis method. This block diagram uses an example pair of the index and middle finger compartments.

Since individual fingers are controlled by separate compartments of the EDC muscle^27^, selecting the precise location of each compartment was required to record corresponding MU activities of each finger. We located the different compartments based on our earlier high-density EMG study, which provide the information of spatial activation of individual compartments^27^, and identified the appropriate electrode location that would yield a high EMG amplitude for a given finger extension. Then a five-pin sensor array (Delsys Inc., Natick, MA) is secured on the skin surface above a compartment. The EMG recordings from one sensor were considered to originate from a single compartment, given that the sensor array tends to give very selective EMG recordings from nearby muscle fibers based on our concurrent sensor array and intramuscular recordings^28^. Specifically, if the intramuscular wire electrodes were inserted outside of the sensor array area spanned by the 5 pins, or if the wire was inserted deep to the muscle, the surface and wire electrodes could not record common MUs. In addition, the obtained action potential duration is also relatively narrow compared with the ones from the highly selective intramuscular electrodes, indicating that only a limited number of superficial fibers were recorded. These findings provide the evidence that the sensor array was selective enough to only record a small portion of the muscle. Since the locations of the two compartments which control the ring and little fingers were not readily distinguishable from the skin surface based on our previous study^27^, we combined the recordings of the ring and little fingers together. The rectangular black boxes in Fig. 1 show the locations of the three compartments (index, middle, and ring-little) of the EDC muscle. In our study, two electrode arrays were used to concurrently collect EMG signals on a pair of separate compartments at any given time. Therefore, the electrode arrays were removed and reattached to different compartments, in order to obtain concurrent MU activities of three possible combination pairs (index vs. middle, index vs. ring-little, and middle vs. ring-little).

#### Experimental Setup & Procedures

Subjects were seated in a straight-back chair with their upper arm comfortably resting on a table, their right shoulder abducted at approximately 30°, the elbow angle extended at approximately 120°, the wrist in neutral position with 0° flexion/extension, and the metacarpophalangeal joint extended at approximately 120°. During the experiment, subjects were instructed to produce isometric finger extensions against a vertical board using all of their four fingers concurrently. A single-differential bar electrode (Delsys Inc., Natick, MA) placed on the mid-belly of the EDC was used to estimate the overall level of EDC activations, and was remained on the same location throughout the testing session, and the RMS value calculated using a 0.8 s moving window was provided to the subject as a feedback for the desired muscle contraction levels. The skin above the EDC muscle was scrubbed with alcohol pads to improve the electrode contact with the skin. Two five-pin sensor arrays (Delsys Inc., Natick, MA) were placed on the different compartment locations as described in the previous section. Five 0.5 mm diameter cylindrical probes were located at the four corners and the center of a 5 × 5 mm square. The electrode array recorded four channels of single differential EMG signals (Fig. 1). The sEMG signals were amplified by a gain of 1000, with a bandwidth of 20 Hz to 500 Hz, and sampled at 20 kHz.

The experiment consisted of two blocks. In the first block, subjects performed maximum effort of finger extension for three seconds, and the resultant RMS value of the EMG from the bar electrode was recorded. The subjects were asked to repeat the maximum effort three times with 60 s rest between trials, and the largest RMS value was then used to estimate the maximum EDC activation. In the second block, five isometric finger extensions (using four finger concurrently) were performed by the subjects. The RMS trace of EMG as in the first block was used as a feedback for the subject to track a target at 50% of the maximum RMS. The EMG recordings from the two sensor arrays were instantaneously shown to the operator. If the operator found that the subject was using one muscle compartment to compensate another, the subject was asked to repeat the trial. Specifically, the subjects were instructed to slowly increase their effect to reach the target and were then asked to maintain their RMS value at the prescribed level for 12 s. A 60 s rest period was provided between each successive trial to avoid cumulative muscle fatigue. The second block was repeated three times, in which EMG signals were obtained from the three different muscle compartment combinations. Therefore, a total of 72 (3 repeated trials ×3 compartment combinations × 8 subjects) trials were collected in this project with two sensor recordings in each trial. All the data were obtained within a single session in the same day for each subject.

### Data Analysis

#### Pre-processing

To obtain reliable EMG decomposition, the data were selected based on the following criteria: (1) the variability of RMS value during steady state contraction was within 10% of the target effort, and (2) the background noise of the sEMG during resting period was within ± 20 µV. Three of the best trials for each finger combination per subject were selected for further analysis.

#### MU Decomposition

Single MU activities were automatically decomposed from the selected sEMG recordings (each array were decomposed separately) using Nawab’s algorithm^26^. The decomposition algorithm extracted the firing times and four different MU action potential waveforms corresponding to the four sEMG channels for each identified MU. The accuracy of the decomposition has been verified using a simulation-based surrogate analysis^29^ and a two-source cross-validation method in which concurrent surface and intramuscular recordings were obtained and decomposed using independent algorithms^28^. An average accuracy of 95% was found in the surface decomposition. Furthermore, a spike triggered average (STA) method was performed to select MU action potential trains based on the stability of the STA estimated waveform as described in a previous study^30^. The validity of the STA method has been assessed using simulated sEMG signals^31^. Specifically, the identified firing times for each MU were used as triggering events for the STA calculation, and the STA was performed on each of the four channels of the EMG signals resulting in four action potentials for each MU. Two tests were then performed: (1) the correlation between the STA estimates and the decomposition templates, and (2) the waveform stability of the STA estimates. The stability of the STA was quantified by the coefficient of variation of the peak-peak amplitude of the waveform over time. A lower variation of the waveform amplitude is regarded as an indicator of more reliable estimate. These tests were designed to assess the stability of the waveform over the trial duration and the degree of match with the decomposition estimated templates. When performed concurrently on four channels of the EMG signals, these tests provided some confidence regarding the reliability of the decomposed MUs^30^.

### MU Coherence Analysis

In this study, three different coherence values— the total coherence, the cross-compartment coherence and the residual of partial coherence were quantified. Specifically, the total coherence measured the input unique to the compartment and the shared common input with other compartments. The cross-compartment coherence only measured the shared common input between two different compartments, and the partial coherence residual represented the input after the cross-compartment coherence was subtracted from the total coherence. The overall block diagram of the coherence calculation is shown in Fig. 1 Part 3.

**Figure 2:**
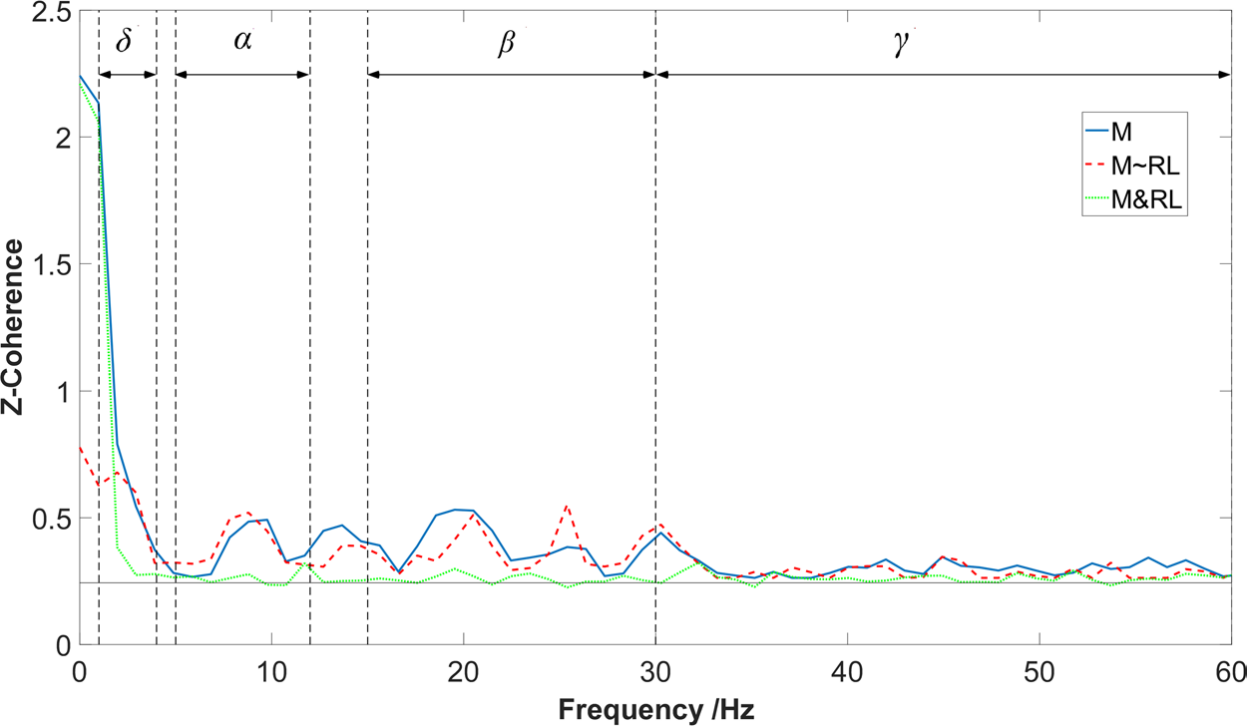
An example of the different coherence calculations from a representative subject. The solid blue line represents the total coherence of middle finger (*M*); the red dashed line represents the partial coherence residual of middle finger after removing any synchrony generated from ring-little finger (*M* ~ *RL*); and the green dotted line represents the cross-compartment coherence of middle and ring-little fingers (*M&RL*). The horizontal line shows the 95% confidence interval.

**Figure 3:**
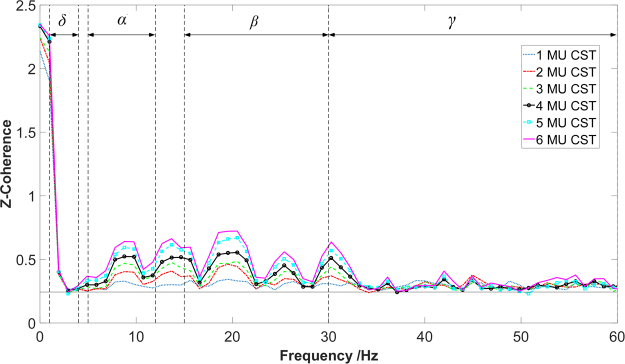
An exemplar of coherence estimate for the number of spike trains vs. magnitude of coherence. Different lines present different number of MUs. MU CST represents motor unit composite spike train. Note: the example trial with the most ripples was selected in order to show the monotonically increasing tendency of the coherence values that were above the baseline, as the number of motor units increased.

To obtain a precise coherence evaluation, the composite spike train (CST) was formed by superimposing the timing of discharge events from multiple MU spike trains (see Fig. 1 from part 2 to part 3), and was used for MU coherence analysis (Fig. 2), as recommended from previous studies^11,12^. The composite spike train results in a substantial increase of the coherence magnitude with an increase of the number of cumulative MU spike trains as shown in Fig. 3, providing more reliable estimates^32,33^. This procedure, however, also requires all coherence calculations to be performed across an equal number of MUs, so that the coherence values can be compared across different conditions^32,33^. Therefore, we selected six MUs randomly from the available MU pool for all the coherence calculation. Previous studies have shown that six MUs are sufficient for a reliable estimate of the coherence^32^.

#### Total and Cross-compartment Coherence Analysis

For the total coherence calculation, a total of six MUs were randomly selected from one muscle compartment, and were randomly separated into two groups with three MUs in each group. The spike timings of the MUs within each group were superimposed to form the CST. The Welch’s averaged, modified periodogram method^34^ was used to calculate the magnitude of the squared coherence ${C}_{{xy}}(f)$ between the two CSTs:

1 $${{\boldsymbol{C}}}_{{\boldsymbol{x}}}{\boldsymbol{y}}({\boldsymbol{f}})|=\frac{{|{{\boldsymbol{P}}}_{{\boldsymbol{x}}}{\boldsymbol{y}}({\boldsymbol{f}})|}^{{\bf{2}}}}{{{\boldsymbol{P}}}_{{\boldsymbol{xx}}}({\boldsymbol{f}}){{\boldsymbol{P}}}_{{\boldsymbol{yy}}}({\boldsymbol{f}})}$$

where ${P}_{{xy}}(f)$, ${P}_{{xx}}(f)$ and ${P}_{{xx}}(f)$ are respectively the cross-spectrum of two CSTs and their auto-spectrum densities. The MATLAB function “mscohere” with a length of 1024 sample segments tapered by a Hann window with 75% overlapped was used for the calculation based on a previous study^35^. For trials with more than six MUs, 100 repetitions of the described procedures were performed, and the coherence values were averaged to reduce potential sampling bias.

For the cross-compartment coherence calculation, the random resampling procedure was similar to the total coherence calculation. However, instead of selecting all of the six MUs from one compartment, three MUs were randomly selected from each of the two compartments for the cross-compartment coherence calculation. The three MU spike trains were then summed up into one CST, and then the cross-compartment coherence was estimated between the two CSTs from the two compartments.

#### Partial Coherence Residual Analysis

To remove the common input from another compartment of the EDC muscle, a well-established algorithm called partial coherence residual analysis has been introduced^25^. This method evaluates the coherence between two CSTs, *x* and *y*, after eliminating the coherence component from a third “reference” signal *z* ^24,36,37^. Specifically, the auto-spectra and cross-spectra between two CSTs *x* and *y*, after accounting for the synchrony of *x* or *y* with reference signal *z*, were calculated as follows:

2 $${{\boldsymbol{P}}}_{{\boldsymbol{xx}}-{\boldsymbol{z}}}({\boldsymbol{f}})={{\boldsymbol{P}}}_{{\boldsymbol{xx}}}({\boldsymbol{f}})-{{\boldsymbol{P}}}_{{\boldsymbol{xz}}}({\boldsymbol{f}}){{\boldsymbol{P}}}_{{\boldsymbol{zx}}}({\boldsymbol{f}})/{{\boldsymbol{P}}}_{{\boldsymbol{zz}}}({\boldsymbol{f}})$$

3 $${{\boldsymbol{P}}}_{{\boldsymbol{yy}}-{\boldsymbol{z}}}({\boldsymbol{f}})={{\boldsymbol{P}}}_{{\boldsymbol{yy}}}({\boldsymbol{f}})-{{\boldsymbol{P}}}_{{\boldsymbol{yz}}}({\boldsymbol{f}}){{\boldsymbol{P}}}_{{\boldsymbol{zy}}}({\boldsymbol{f}})/{{\boldsymbol{P}}}_{{\boldsymbol{zz}}}({\boldsymbol{f}})$$

4 $${{\boldsymbol{P}}}_{{\boldsymbol{xy}}-{\boldsymbol{z}}}({\boldsymbol{f}})={{\boldsymbol{P}}}_{{\boldsymbol{xy}}}({\boldsymbol{f}})-{{\boldsymbol{P}}}_{{\boldsymbol{xz}}}({\boldsymbol{f}}){{\boldsymbol{P}}}_{{\boldsymbol{zy}}}({\boldsymbol{f}})/{{\boldsymbol{P}}}_{{\boldsymbol{zz}}}({\boldsymbol{f}})$$

Therefore, the residual coherence between *x* and *y* after eliminating any interference from *z* is:

5 $${{\boldsymbol{C}}}_{{\boldsymbol{xy}}-{\boldsymbol{z}}}({\boldsymbol{f}})=\frac{|{{\boldsymbol{P}}}_{{\boldsymbol{xy}}-{\boldsymbol{z}}}({\boldsymbol{f}})|}{{{\boldsymbol{P}}}_{{\boldsymbol{xx}}-{\boldsymbol{z}}}({\boldsymbol{f}}){{\boldsymbol{P}}}_{{\boldsymbol{yy}}-{\boldsymbol{z}}}({\boldsymbol{f}})}$$

In this study, *x* and *y* represents the two CSTs with three MU spike trains each from a particular muscle compartment, and *z* represents the CST with the sum of all MU spike trains from a different compartment.

#### Coherence of Each Frequency Bandwidth

The squared coherence over different frequency ranges was divided into four different frequency bands— the delta band (1–4 Hz), the alpha band (5–12 Hz), the beta band (15–30 Hz), and the gamma band (30–60 Hz), with each bandwidth represents different physiological meanings^5,13,15^. The magnitude of the coherence for each band was calculated by averaging the coherence^21^:

6 $${\boldsymbol{Mean}}\,{\boldsymbol{Coherence}}=\frac{{\int }_{{{\boldsymbol{f}}}_{2}}^{{{\boldsymbol{f}}}_{2}}{\boldsymbol{C}}({\boldsymbol{f}}){\boldsymbol{df}}}{{\boldsymbol{B}}}$$

where $C(f)$ is the magnitude of squared coherence, B is the width of one frequency band, and ${f}_{1}$ and ${f}_{2}$ are the lower and upper bounds of the corresponding band. The confidential level of the coherence estimate was calculated:

7 $${{\boldsymbol{\gamma }}}_{{\bf{1}}-{\boldsymbol{\alpha }}}^{{\bf{2}}}={\bf{1}}-{{\boldsymbol{\alpha }}}^{{\boldsymbol{[}}1{\boldsymbol{/}}{\boldsymbol{(}}1{\boldsymbol{/}}{\boldsymbol{DOF}}-1{\boldsymbol{)}}{\boldsymbol{]}}}$$

where $\alpha$ is (1 − $\alpha$)% confidence level, ${DOF}$ is the degree of freedom. The ${DOF}$ was calculated as $(8/3)(N/M)$ for Hann window^38^, where $N$ is the number of data points in the time series and $M$ is the half-width of the window in the time domain.

Based on several previous studies, the coherence calculation always has an intrinsic baseline value^21,25^, which was typically removed from the raw coherence values before further analysis. This baseline bias was empirically eliminated by subtracting the coherence of each frequency bandwidth by the average coherence calculated between 300 and 500 Hz, which is believed to contain no physiological information^39^. The range of this intrinsic baseline was approximately from 0.03 to 0.04 (the corresponding z-coherence values were 0.175 to 0.203). Even if two CSTs with no common input were used for coherence calculation, the mean value of the coherence was still not zero. This level of bias is determined by the number of data segments used for coherence calculation^40^.

Some notations were introduced to simplify the description of our results. Specifically, *I*, *M*, and *RL* are used to represent the index, middle and ring-little, respectively. “~” means removal of the common input arisen from another compartment, and “*&*” signifies the cross-compartment coherence between two compartments. For example, “*I~M*” is the partial coherence residual of the index finger after removing common input from the middle finger; “*I&M*” is the cross-compartment coherence between index and middle fingers. An exemplar coherence estimation of the total, partial and cross-compartment coherence is shown in Fig. 2.

#### Statistics

Our analyses focused on three aspects: the four frequency bands (delta, alpha, beta, and gamma bands), the three types of coherence (total, cross-compartment, and partial residual), and the three finger combinations (*I* vs. *M*, *I* vs. *RL* and *M* vs. *RL*). Prior to the statistical analysis, the coherence values of the three repeated trials of each condition were averaged to improve the coherence estimates. All statistical analyses were performed in SPSS (version 24. IBM Corp., Armonk, NY). Repeated measures ANOVAs were performed to investigate these factors. A significance level of *p* < 0.05 was used. When necessary, *post hoc* pairwise comparisons were conducted with Bonferroni correction (the *p* value was adjusted to 0.05 divided by the total number of pairwise comparisons). In addition, to satisfy the ANOVA assumption, the Fisher’s *z*-transformation was applied to convert all coherence values to Fisher’s value, which has been successfully used in previous studies^11,21,41,42^. The equation of Fisher’s *z*-transformation is:

8 $${\boldsymbol{Z}}-{\boldsymbol{Coherence}}={\bf{arctn}}{\boldsymbol{h}}\sqrt{{\boldsymbol{C}}({\boldsymbol{f}})}$$

### Data Available

The datasets generated during and/or analyzed during the current study are available from the corresponding author on reasonable request.

## Results

A total of 144 (3 trials × 2 arrays × 3 compartment combinations × 8 subjects) EMG recordings were decomposed. The average number of accepted MUs was 16.55 ± 6.10 per trial. First, we began by examining the influence of the number of MUs on the coherence calculation. Although a larger number of spike trains provided a stronger coherence, the increasing tendency was still consistent across different bandwidths^32^ (see Fig. 3 as an exemplar calculation based on the total coherence of a single trial). This monotonic increase in the coherence values was also a common observation across the different trials. Since all coherence values (that were above the baseline) increased uniformly across different frequency bandwidths, as the total number of motor unit increased, the general statistical outcomes should not be changed. Due to the small number of accepted MU spike trains in several trials, a total of six spike trains (three for each CST) were selected to ensure a good coherence estimate across different conditions.

Second, we investigated the potential difference between cross-compartment coherence and partial residual coherence under each compartment combinations and across the four frequency bandwidths. Three separate two-way (frequency band × coherence type) repeated measures ANOVAs were performed on the different finger combinations.

### Index vs. Middle

**Figure 4:**
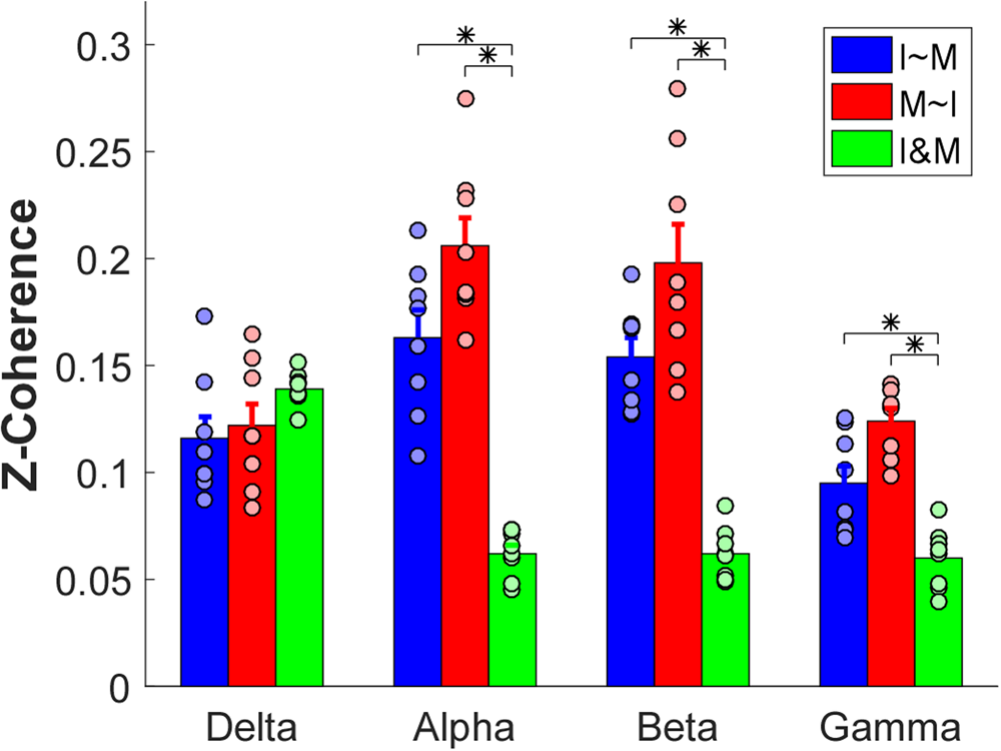
Overall comparisons across two partial residuals and cross-compartment coherence in the index vs. middle combination. Each bar represents the average magnitude of z-coherence, and the error bars represent standard error across subjects. Asterisks indicate statistical significance. The black circles represent values of individual subjects. Different colors represent the different types of coherence.

The ANOVA showed a significant interaction [F(6,42) = 20.203, *p* < ${10}^{-10}$] between frequency band and coherence type. Further pairwise post hoc comparisons were tested to compare the difference of coherence type in each frequency bands (Fig. 4). For the delta band, the two partial residuals (*I~M* and *M~I*) and the cross-compartment coherence showed no significant difference (*p* > 0.05). For the rest of the three bands, the two partial residuals maintained at the same level (*p* > 0.05), and both were higher than the cross-compartment coherence (*p* < 0.05).

### Index vs. Ring-Little

**Figure 5:**
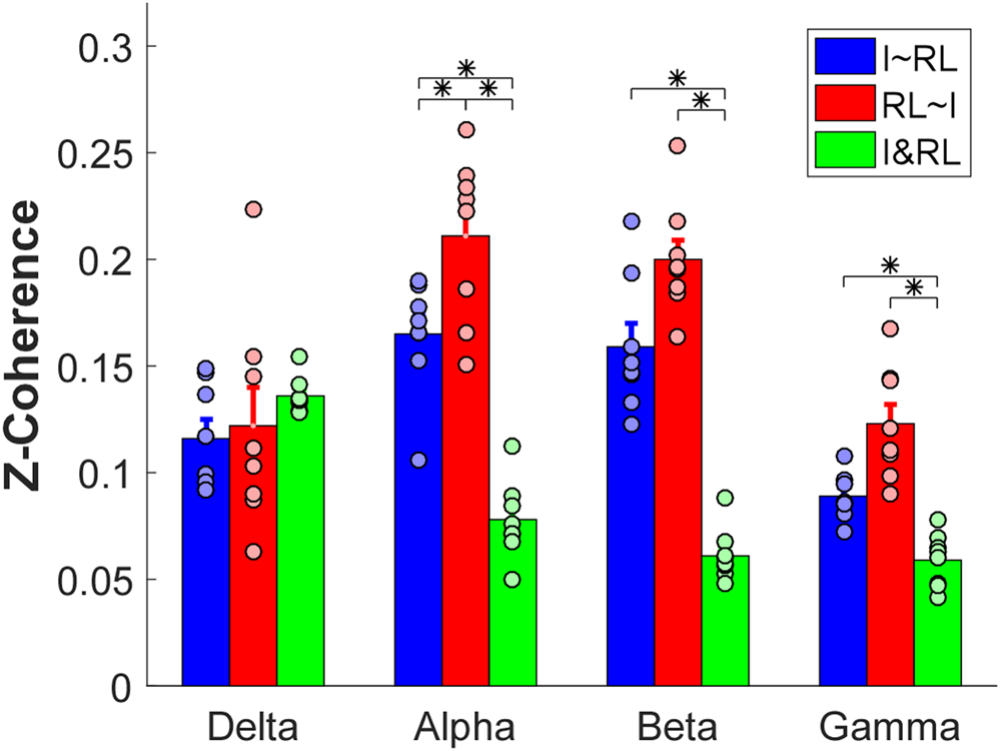
Overall comparisons across two partial residuals and cross-compartment coherence in the index vs. ring-little combination. Each bar represents the average magnitude of z-coherence, and the error bars represent standard error across subjects. Asterisks indicate statistical significance. The black circles represent values of individual subjects. Different colors represent the different types of coherence.

The ANOVA results showed a significant interaction [F(6,42) = 22.480, *p* < ${10}^{-10}$] between frequency band and coherence type. Further post hoc pairwise comparisons were performed in each frequency band (Fig. 5). For the delta band, the two partial residuals (*I~RL* and *RL~I*) and the cross-compartment coherence showed no significant difference (*p* > 0.05). For the alpha band, *RL~I* was higher than *I~RL* (*p* = 0.049), and both of the two partial residuals was larger than the cross-compartment coherence (*p* < 0.05). For the rest of two bands, the two partial residuals showed no significant difference (*p* > 0.05), and both were higher than the cross-compartment coherence (*p* < 0.05).

### Middle vs. Ring-Little

**Figure 6:**
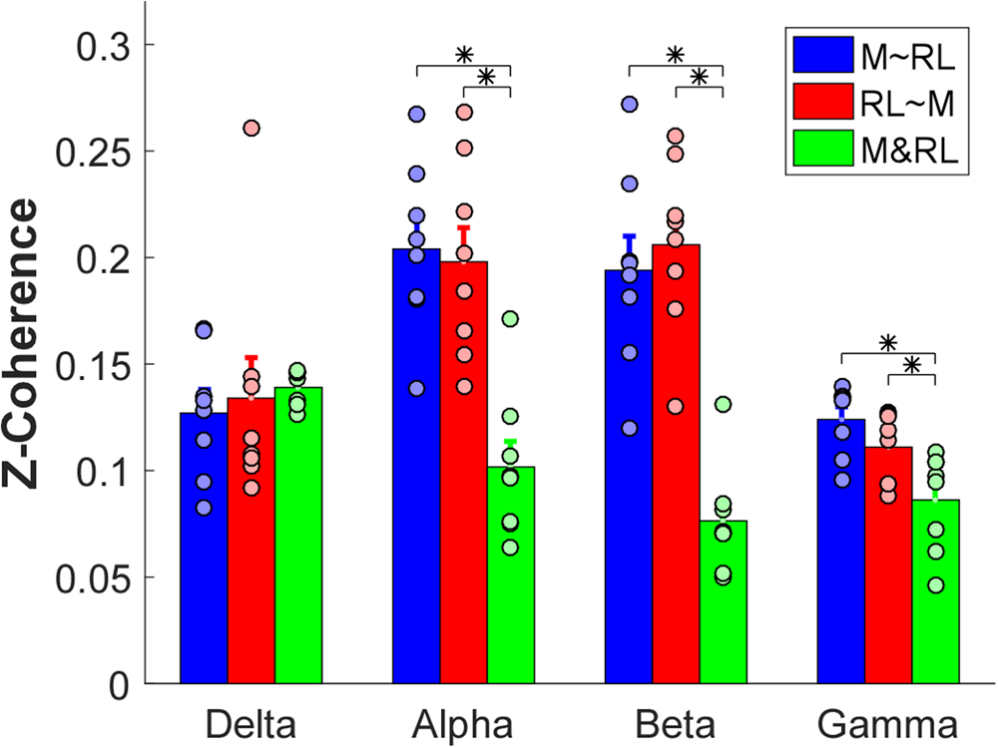
Overall comparisons across two partial residuals and cross-compartment coherence in the middle vs. ring-little combination. Each bar represents the average magnitude of z-coherence, and the error bars represent standard errors. Asterisks indicate statistical significance. The black circles represent values of individual subjects. Different colors represent the different types of coherence.

The two-way ANOVA showed an interaction [F(6,42) = 12.744, *p* < ${10}^{-7}$] between frequency band and coherence type. The post hoc tests showed that (1) the three types of coherence had no significant difference in the delta band (*p* > 0.05); (2) two partial residuals *M~RL* and *RL~M* were at the similar level (*p* > 0.05), and both were significantly larger than (*p* < 0.05) the cross-compartment coherence in the rest of three bands (Fig. 6).

**Figure 7:**
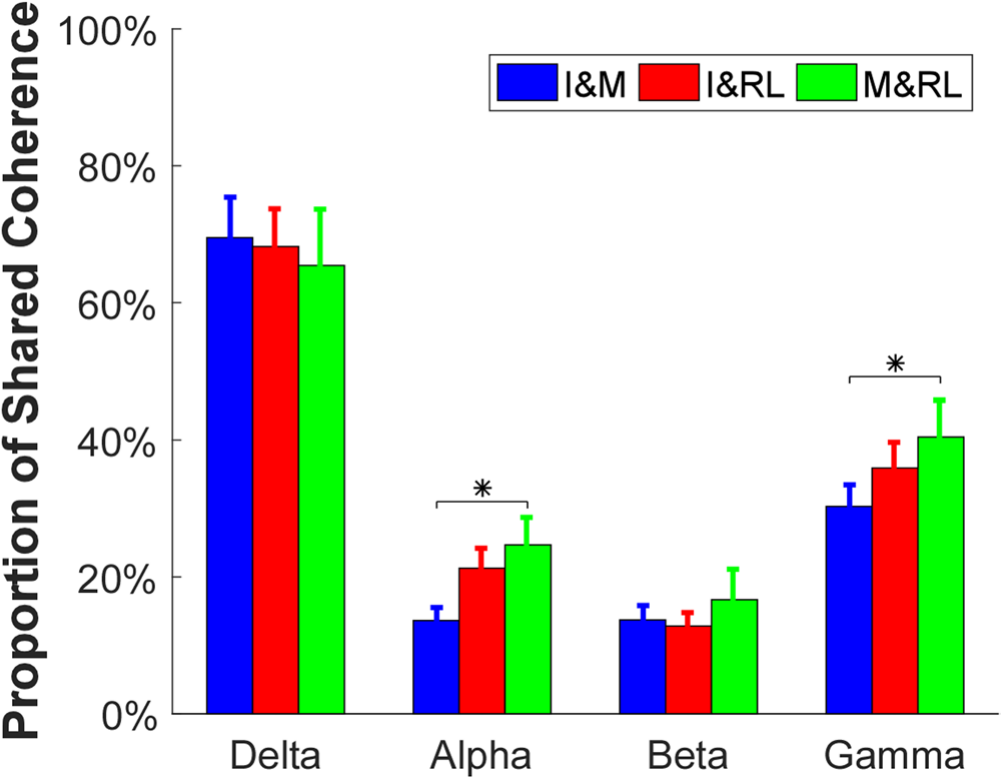
Proportion of cross-compartment z-coherence to its corresponding total z-coherence. Each bar represents the average proportion, and the error bars present the standard error across subjects. Asterisks indicate statistical significance. Different colors represent different types of coherence.

We also evaluated the proportion of the cross-compartment z-coherence relative to the total z-coherence across the three finger combinations (Fig. 7). The two-way ANOVA (frequency band × finger combination) revealed a significant interaction [F(6,42) = 2.748, *p* = 0.03]. The further pairwise post hoc comparison showed that (1) the ratio in the delta band was significantly higher than any other band (*p* < 0.05) for all the three finger combinations; (2) the ratio of the three finger combinations had no significant difference in the delta band (*p* = 1); (3) the ratio of the *M*&*RL* combination was higher than *I*&*M* in the gamma band (*p* < 0.05); and (4) the ratio of *M*&*RL* was higher than *I*&*M* in the alpha band (*p* = 0.03).

## Discussion

The purpose of this study was to evaluate the level and the different origins of shared common input to the different compartments of the EDC muscle. A coherence analysis of MU discharge events was used to quantify the relative contribution of shared vs. separate neural input to the different compartments, and the different origins of common input can also be characterized based on the coherence level in each previously defined frequency bandwidth. Our results reveal a low level (<20% of the total common drive) of shared input to different compartments of the EDC in the alpha and beta bands, a moderate (30–40% of the total common drive) level of shared input in the gamma band, and a high (>60% of the total common drive) level of shared input in the delta band, admittedly the definition of the different levels was rather arbitrary. The results also show that the level of shared input tends to be higher between the compartments that control the middle finger and ring-little fingers. Our findings indicate that the level of synchronization across different compartments of the EDC muscle tends to vary depending on the origins of the shared input. Overall, through a systematic evaluation, the outcomes of our study provide detailed evidence regarding the synchronized activation of the EDC muscle, which also provide baseline information for understanding potential changes in muscle synchronization/coordination of individuals with neuromuscular disorders.

Earlier studies have found a moderate level of synchronization among the different compartments of the finger extensor and flexor muscles, although the temporal synchronization of MU discharge within a compartment still dominate the overall level of synchrony^10^. Consistent with these observations, our results further extended those earlier findings and showed that the level of shared common input across different compartments varies depending on the specific frequency bandwidths; namely, the different levels of the central nervous system contributed differently on the coordinated activation of the EDC muscle compartments. Specifically, we found that the shared common input (as quantified by the cross-compartment coherence) in the delta band was comparable to the common input within individual compartments, and this is the case in all our tested finger combinations. Our findings are consistent with a recent study, which reported that the MU pools of different human muscles (abductor digiti minimi, tibialis anterior and vastus medialis) received a similar and large (>60%) proportion of the common input with respect to the total input at the low frequency band (<5 Hz). It is known that the coherence in the low frequency common input as in delta band is associated with common modulation of MU mean firing rate and with the generation of muscle force^16^. The amount of low frequency common input also constitutes a substantial portion of the total synaptic input^43^. Common input at relatively higher frequencies may have little influence on the muscle force output, due to low-pass filtering effect of the muscle^44^, but these high frequency oscillations can still reflect the effective higher level neural linkage among muscles and can facilitate different parallel and hierarchical control structures^45,46^. Our findings indicate that the overall mean firing rates of MUs both within a compartment and across compartments are modulated in a coordinated manner. However, this effect could arise from our testing paradigm in that the subjects were instructed to produce concurrent four-finger extensions, which was required to obtain sufficient concurrently active MUs from different compartments for the coherence calculation. Future experiment that involves un-coordinated finger extension is necessary to evaluate the cross-compartment coherence in the delta band when the firing rate may not be modulated concurrently.

We also found that the shared input in the alpha band had minimal contribution to the overall synchronization across compartments. The coherence in the alpha band is thought to originate from spinal reflex input^17,18^. For example, earlier studies have shown an increased coherence in the alpha band when the muscle performed dynamic contractions with substantial afferent inputs from muscle spindles, in comparison with the isometric contraction conditions^47^. Our findings suggest that the reflex circuitry in the EDC muscle is compartmentalized; namely, the spindle afferent of a particular compartment primarily project to the motor neurons controlling the same muscle compartment. Although reflex compartmentalization based on muscle mechanical actions has been demonstrated in cat models^48^, direct evidence on the extent of reflex segregation in the EDC muscle is lacking. Further investigation that can quantify reflex activation of non-stretched fingers is needed to confirm our findings. Similarly, a low level of cross-coherence in the beta band, with subcortical to cortical origins, is also observed across different finger combinations. These results indicate that spinal and supraspinal circuitry are not primary factors that can contribute to the synchronization across different muscle compartment in healthy individuals. On the other hand, earlier studies in animal models and stroke survivors^4,49–51^ have shown that a lesion in the cortical regions and the corticospinal tract can substantially increase synchronized activation of finger muscles. Therefore, we expect to observe increased cross-compartment coherence in the alpha and beta bands in chronic stroke survivors in future studies, and the findings from our current study can provide normative baseline for studies involving clinical populations.

A recent study has shown that the synergistic knee extensors shared a majority of the common input during isometric knee extensions^25^. However, we only reported a low to moderate level of shared common input in the different compartments of the EDC muscle. These different findings could largely arise from the different functionality of the muscles involved. The previous study tested the thigh muscles and these two synergistic knee extensor muscles are activated concurrently during knee extension, and a strong shared input is beneficial for synchronized knee torque generation and simplified control of muscle activation. However, the EDC muscle controls all four-finger extensions, and individualized activation of specific compartments is required during dexterous finger movement. Therefore, a relatively low level of shared input will allow selective extension of individual fingers in a majority of the daily activities.

Despite a low to moderate level of shared input among different fingers, we still found that the shared common input between the compartments controlling the middle and ring-little fingers is stronger than the shared input between other finger combinations, and the index finger exhibits the lowest level of shared input with other fingers, demonstrating the highest degree of independence. Our findings are consistent with previous studies that quantified the coordinated movement of fingers based on joint kinematics^52^, EMG amplitude^4^, and MU synchronization^10^. Our results show that the coherence in the alpha and gamma bands is significantly higher between the middle and ring-little finger combination. These findings indicate that the relatively high synchronization between the compartments controlling middle and ring-little fingers arises from the spinal and cortical levels. Admittedly, we combined the little and ring compartments during the coherence calculation, largely due to the fact that MUs from these two compartments are not securely distinguishable from the skin surface^27^. In comparison with the strictly pairwise comparisons, our procedure could inevitably increase the estimated cross-compartment coherence by pooling the MUs from two compartments.

In our experiment, only two separate sets of MU activities were obtained concurrently from two sensor arrays. Although we can reliably estimate the shared common input based on the cross-compartment coherence calculation, the separate common input that is unique to each compartment cannot be accurately estimated. Therefore, the term ‘partial coherence residual’ rather than the ‘unique coherence’ was used in the study. Essentially, the separate input based on the partial coherence residual would be higher than the unique coherence value specific to a particular compartment, because the residual still has a proportion of coherence that is shared with other compartments and even with other muscle groups. We could reduce the estimation bias by recording the MU activity of all the EDC compartments concurrently via other EMG acquisition approaches, such as high-density EMG. The estimated coherence may still not be strictly unique, because of potential co-activation of EDC with muscles controlling wrist movements. In addition, although the sensor arrays are selective, different arrays could still detect the same MUs, which would lead to overestimated cross-coherence values. Based on our estimated coherence values, we believe that the chances of recording the same MU should be low, otherwise we would observe uniformly high coherence not modulated at different frequency bandwidths, if the two composite spike trains were coming from duplicate MU firing spike trains.

In conclusion, we quantified the relative level of shared common input across different compartments of the EDC muscle and also characterized the different origins of the common input. Our results show that the overall level of shared input ranges from low to high levels, and the specific level of shared input varies depending on the sources of the common input. Our findings provide detailed information regarding the neural component of coordinated finger muscle activation, and also provide base line information for studies that investigate the change of synchronized activation of finger muscles in individuals with neuromuscular disorders.
